# Coherent Electron–Phonon Coupling in Two-Dimensional Bi_2_Se_3_ Nanoplatelets Studied with Ultrafast Spectroscopy

**DOI:** 10.1021/acs.jpcc.6c00732

**Published:** 2026-04-15

**Authors:** Alessandro Baserga, Jara F. Vliem, Rhea Kumar, Riccardo Reho, Andrés R. Botello-Méndez, Daniel Vanmaekelbergh, Zeila Zanolli, Giulio Cerullo

**Affiliations:** † Department of Physics, 18981 Politecnico di Milano, 20133 Milan, Italy; ‡ Debye Institute for Nanomaterials Science, 8125 Utrecht University, Princetonplein 1, 3584 CC Utrecht, The Netherlands

## Abstract

Electron–phonon coupling in two-dimensional Bi_2_Se_3_ nanoplatelets was investigated using broadband ultrafast pump–probe spectroscopy (probe 1.6 to 2.8 eV) at 293 and 78 K. The high time resolution resolves coherent phonon oscillations on picosecond time scales, specifically the out-of-plane A_1g_^(1)^ (2.12 THz) and A_1g_^(2)^ (4.95 THz) optical modes, and a low-frequency interlayer breathing mode (∼0.4 THz), arising from standing waves defined by the nanoplatelet thickness. By mapping the probe energy dependence of the oscillation amplitude, we find that the A_1g_^(1)^ mode couples most strongly to an electronic transition near 1.97 eV, which we assign using the computed band structure to transitions along the high-symmetry Γ–K line. The absence of Raman-active E_g_ modes is explained by a symmetry analysis based on the displacive excitation of coherent phonons. These results characterize carrier–lattice interactions in 2D Bi_2_Se_3_, which are relevant for optoelectronic device applications.

## Graphical abstract

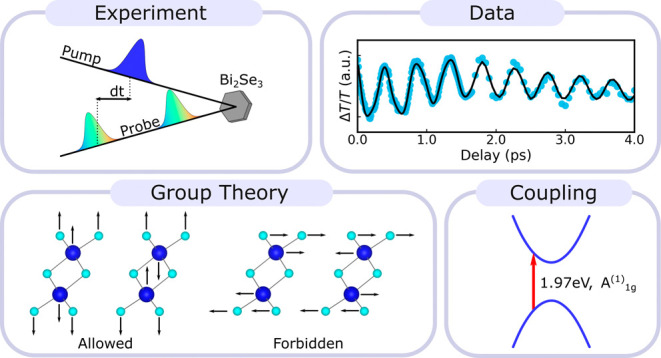

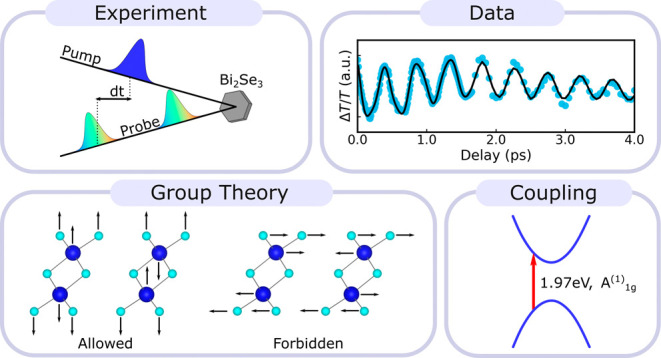

## Introduction

Bismuth selenide (Bi_2_Se_3_) is a widely studied thermoelectric converter,^1–4^ optoelectronic material,^5–10^ and topological insulator (TI).^11,12^ It crystallizes in the *D*_3d_ point group, and its structure consists of stacked Se–Bi–Se–Bi–Se quintuple layers (QLs), separated by van der Waals gaps. When the number of QLs is above 7, Bi_2_Se_3_ is considered a three-dimensional TI, which is insulating in the bulk but has protected spin-momentum locked surface states.^11^ In thinner 2D films, hybridization between the protected surface states opens a gap between these states, and the gapped states are integrated into the band structure.^13,14^ This mixing of surface and bulk states results in unusual optical transitions and carrier relaxation pathways.^14,15^ Consequently, understanding electron–phonon coupling in 2D Bi_2_Se_3_ is required to balance carrier mobility, lifetime, and thermal transport, as these factors determine the efficiency of optoelectronic and thermoelectric devices.^16–20^

For Bi_2_Se_3_, the optical phonon modes (in-plane E_g_^(1)^, E_g_^(2)^ modes, and out-of-plane A_1g_^(1)^, A_1g_^(2)^ modes, see Figure 1) have been identified with Raman spectroscopy.^21–23^ Previous ultrafast transient absorption studies have explored carrier dynamics in bulk^24–27^ and thin films,^7,14,28,29^ with some reports focusing on coherent acoustic phonons^30–32^ and the lower-frequency A_1g_^(1)^ mode.^33,34^ Several reports have also focused on disentangling the specific contributions from surface and bulk states to these oscillations in 2D and 3D Bi_2_Se_3_, with most reports agreeing that coupling to bulk states dominates in 3D Bi_2_Se_3_ while coupling to surface states may be enhanced in thinner crystals.^23,35–38^ Generally, the interaction between phonon modes and photoexcited charge carriers, particularly in the thin-film limit, is complex and depends on film thickness or surface effects.^21,23,37,39^ For example, the A_1g_^(1)^ mode shows frequency softening (i.e., a redshift) as the sample thickness decreases below 15 QL, which is attributed to a reduction in the interlayer van der Waals forces.^21^ Despite these reports, it remains unclear which states are exactly involved in electron–phonon coupling.^23,35–38^

**Figure 1:**
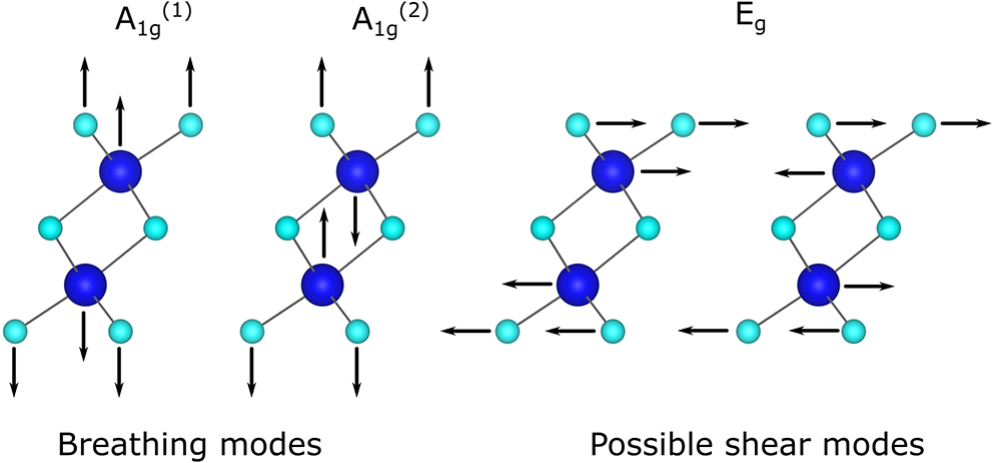
Phonon modes in bismuth selenide. Schematic showing the phonon modes in bismuth selenide. The structure of Bi_2_Se_3_ was obtained from the inorganic crystal structure database with collection code 9011965 ^42^ and visualized using the VESTA software.^43^

Here, we investigate electron–phonon coupling and coherent phonon dynamics in Bi_2_Se_3_ nanoplatelets (NPLs) with well-defined thickness and lateral dimensions. Unlike epitaxial films, these NPLs are freestanding, which eliminates substrate-induced strain and damping.^30,40^ To understand the dynamic interaction between lattice vibrations and photoexcited carriers, we performed ultrafast broadband pump–probe spectroscopy to track coherent lattice oscillations with femtosecond resolution at 293 and 78 K. We observe the dominance of the totally symmetric A_1g_ modes, consistent with a displacive excitation mechanism.^41^ We also resolve a confined acoustic breathing mode (∼0.4 THz), which we used to verify the thickness of our NPLs. Finally, by resolving the probe energy dependence of the oscillations, we demonstrate that the A_1g_^(1)^ mode couples resonantly to electronic transitions at 1.97 eV. Based on the computed band structure of Bi_2_Se_3_, we attribute this coupling to transitions along the high-symmetry Γ–K line. This combination of ultrafast spectroscopy and theoretical modeling can thus be used to identify specific scattering pathways in 2D Bi_2_Se_3_.

## Experimental and Theoretical Methods

### Chemicals

Sodium selenite (Na_2_SeO_3_, 99%), bismuth­(III) nitrate pentahydrate (Bi­(NO_3_)_3_·5H_2_O, 99.999%), polyvinylpyrrolidone (*M*_*w*_ = 10,000), anhydrous ethylene glycol (99.8%), and anhydrous acetonitrile (99.8%) were purchased from Aldrich. Anhydrous ethanol (99.8%) and acetone (99.8%) were sourced from VWR Chemicals. All chemicals were used without further purification.

### Synthesis procedure

Bi_2_Se_3_ NPLs were synthesized as previously described by Moes et al.^13^ The Se precursor was prepared by mixing 0.05 g sodium selenite, 0.22 g polyvinylpyrrolidone (PVP) and 9.5 mL ethylene glycol in a 50 mL round-bottom flask. After degassing for 15 min at room temperature, the solution was heated to 50 °C. Upon reaching 50 °C, the flask was put under N_2_ atmosphere and the mixture was heated further to 190 °C. In the meantime, 0.2 g Bi­(NO_3_)_3_·5H_2_O in 1 mL ethylene glycol was heated under N_2_ on a 200 °C heating plate. Heating of this Bi injection precursor was continued for 30 s after its transition from clear and colorless to turbid white, which occurs around 140 °C. When the Se precursor reached 190 °C, 0.5 mL of the Bi precursor was injected to form Bi_2_Se_3_ platelets, which were left to grow for 10 min. The reaction was cooled to room temperature with a water bath and the black product was washed by adding a mixture of acetonitrile and ethanol, followed by centrifugation. The resulting black precipitate was redispersed in 10 mL ethanol and stored in a glovebox under N_2_ atmosphere.

### Sample Characterization

TEM samples were prepared by drop-casting a diluted dispersion of NPLs on 200 mesh Formvar/carbon-coated Cu TEM grids. The samples were imaged using a Talos F200X (S)­TEM operating at 200 keV. For the side view of the NPL film, TEM lamellae were prepared from Bi_2_Se_3_ nanoplatelets (NPLs) using a FEI Helios G4 Dual Beam equipped with a Ga ion source. To mitigate charging, NPLs were drop-cast onto a conducting silicon wafer. The film was coated with Pt to protect the NPLs from ion-induced damage. After milling, a final cleaning step was performed at 5 kV and 15 pA to remove surface Ga contamination and residues. The sample was then imaged using an aberration-corrected Thermo Scientific Spectra 300 (S)­TEM operating at 300 keV.

UV–vis measurements were performed on a PerkinElmer Lambda 950 UV–vis-NIR spectrometer, using a quartz cuvette with a path-length of 1 cm. The UV–vis samples were prepared by diluting a stock dispersion of NPLs in ethanol approximately 300 times, which yielded an optical density of 1 at the absorption maximum.

For transient absorption measurements, the stock NPL dispersion was dried and redispersed in acetonitrile, after which it was diluted and drop-cast onto a 500 μm UV-fused silica substrate. We used dropcast films in vacuum to avoid oxidation of the samples, and to compare room temperature and low temperature measurements. Furthermore, previous measurements on such samples proved successful.^14^

### Transient Absorption Experimental Setup

The transient absorption setup was driven by a regeneratively amplified Ti:sapphire laser (Coherent Libra) with a fundamental wavelength at 800 nm, pulse duration of approximately 100 fs and a repetition rate of 2 kHz.

The pump pulses were generated by a visible noncollinear optical parametric amplifier (NOPA) pumped by the second harmonic of Ti:sapphire. To generate the pump of the NOPA, a 2 mm β-barium borate (BBO) crystal was used, while the seed was obtained using a 1 mm sapphire crystal. The output pulse was centered at 590 nm with an approximate bandwidth of 120 nm and was compressed down to 20 fs using a pair of chirped mirrors. The pump fluence in the experiments was set to 340 μJ/cm^2^.

To generate the probe, the laser fundamental was focused into a 3 mm thick sapphire crystal, resulting in a broadband pulse with a spectrum ranging between 470 and 780 nm. The pump and probe spectra are shown in Figure S1.

The pump and probe pulse were focused onto the sample, which was placed in a continuous flow cryostat (MicrostatHires, Oxford Instruments) connected by low-loss transfer tube to a liquid nitrogen Dewar and kept in vacuum, using a metallic spherical mirror. After interacting with the sample, the probe was recollimated using a lens and sent to the spectrometer. Unwanted pump photons were filtered by inserting a polarizer after the lens, since the pump and probe pulses were cross-polarized.

The differential transmission signal is defined as

1 $$\Delta T/T=\frac{\left(T_{pump\;on}-T_{pump\;off}\right)}{T_{pump\;off}}$$

where *T* indicates transmission. The differential transmission was recorded using a fast visible camera (Stresing FLCC3001-FFT) coupled to a Princeton Instruments spectrometer as a detector.

### Theoretical Methods and Computational Details

Density Functional Theory (DFT) band structure calculations and the corresponding symmetry analysis were conducted using the QuantumESPRESSO software package.^44,45^ We employed norm-conserving fully relativistic pseudopotentials obtained from PseudoDOJO,^46^ which included spin–orbit coupling (SOC). The Generalized Gradient Approximation (GGA) within the Perdew–Burke–Ernzerhof (PBE) functional was utilized for exchange-correlation. The electronic Brillouin zone was sampled using a 12 × 12 × 1 k-point grid, and a wave function kinetic energy cutoff of 140 Ry was applied. The crystal structure was obtained as detailed in Moes et al.^13^

## Results and Discussion

Hexagonal 2D Bi_2_Se_3_ NPLs with an average thickness of 4.3 ± 1.1 QLs (see Figure 2A for TEM image of the sample and Figure S2 for characterization of nanoplatelets) were excited by a 20 fs pulse centered at 2.1 eV. Due to the random in-plane orientation of the NPLs within the film, the measured response represents an ensemble average over all basal-plane directions (see Figure S3 for a cross-section of a drop-cast film).

**Figure 2:**
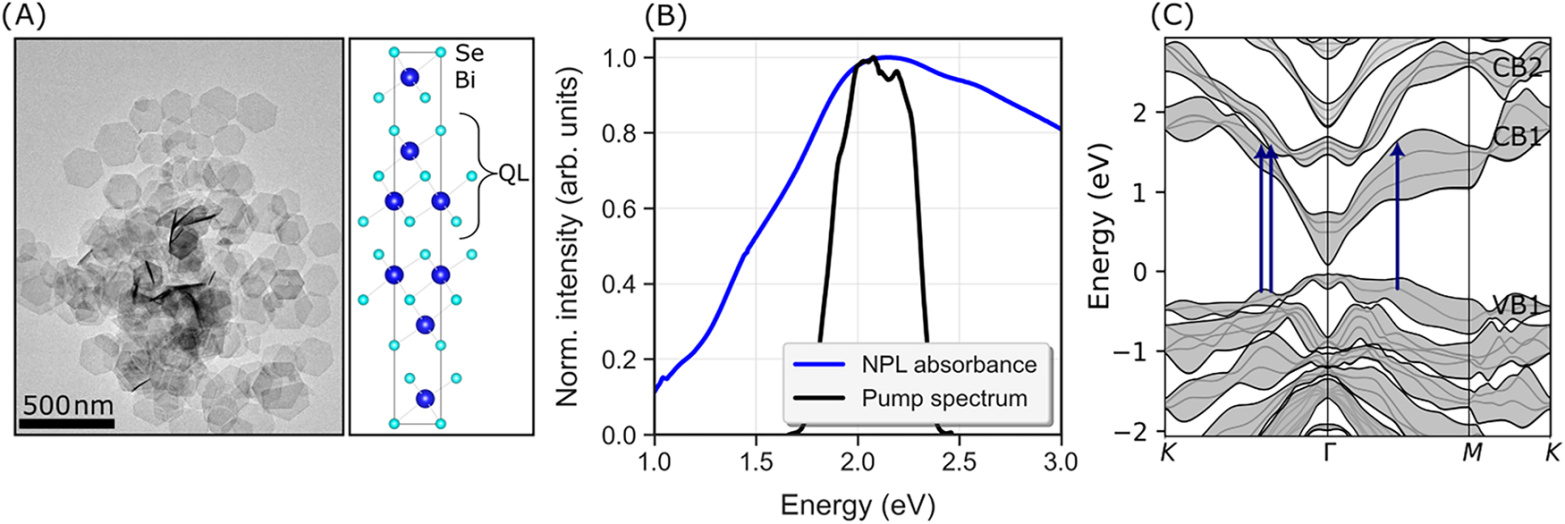
NPLs and pump–probe measurements. (A) TEM image of Bi_2_Se_3_ platelets and the schematic structure of Bi_2_Se_3_ along the *c*-axis. The structure of Bi_2_Se_3_ was obtained from the inorganic crystal structure database with collection code 9011965.^42^ (B) Normalized absorbance of Bi_2_Se_3_ nanoplatelets in ethanol, and the normalized pump spectrum. (C) Schematic of electronic band structure for 4 QL Bi_2_Se_3_. VB1, CB1 and CB2 indicate valence/conduction band manifolds 1 or 2. Possible strong transitions for 1.85 eV (i.e., corresponding to an experimental excitation of 2.1 eV, see Supporting Text) have been drawn in the band structure as blue arrows.

In Figure 2B, the pump spectrum is overlaid with the absorption spectrum, showing a strong optical absorption at the excitation energy used in our pump–probe measurements. Based on density functional theory (DFT) calculations for 6 QL slabs (detailed discussion of DFT results, and validation against optical measurements can be found in Vliem et al.^14^), we identify likely photoexcited transitions at this energy. A brief discussion of this process can also be found in the Supporting Text. The identified transitions are shown in Figure 2C, halfway along the Γ–K line (valence band (VB) 1 to conduction band (CB) 1 and 2 manifolds) and on the line Γ–M (VB1 to CB1 manifolds). Of these, the strongest theoretical absorbance occurs along Γ–K, possibly due to overlapping CB1 and CB2 band manifolds, which results in a relatively high density of states at the transition energy. These states are mostly associated with the inner QLs, not the surface QLs.

In addition to exciting carriers, the pump pulse generates coherent phonons of various frequencies.^41^ These appear in pump–probe measurements as damped oscillations superimposed on a nonoscillatory background from electronic excitation and relaxation. Figure 3A shows the transient transmissivity map Δ*T*/*T*(*h*ν) at 78 K across probe energies from 1.7 to 2.8 eV. Additional data at 293 K, used to verify our low temperature data and to investigate phonon softening, are shown in Figure S4 of the Supporting Information. Figure 3A is dominated by positive signal arising from state filling, specifically a broad band at 1.8 to 2.1 eV, and a secondary peak near 2.65 eV. This is in agreement with prior results.^14^ Figure 3B shows dynamics at several important probe energies, namely at the bleach peak positions (2.05 and 2.60 eV) and at both edges of the broad bleach (1.75 and 2.30 eV).

**Figure 3:**
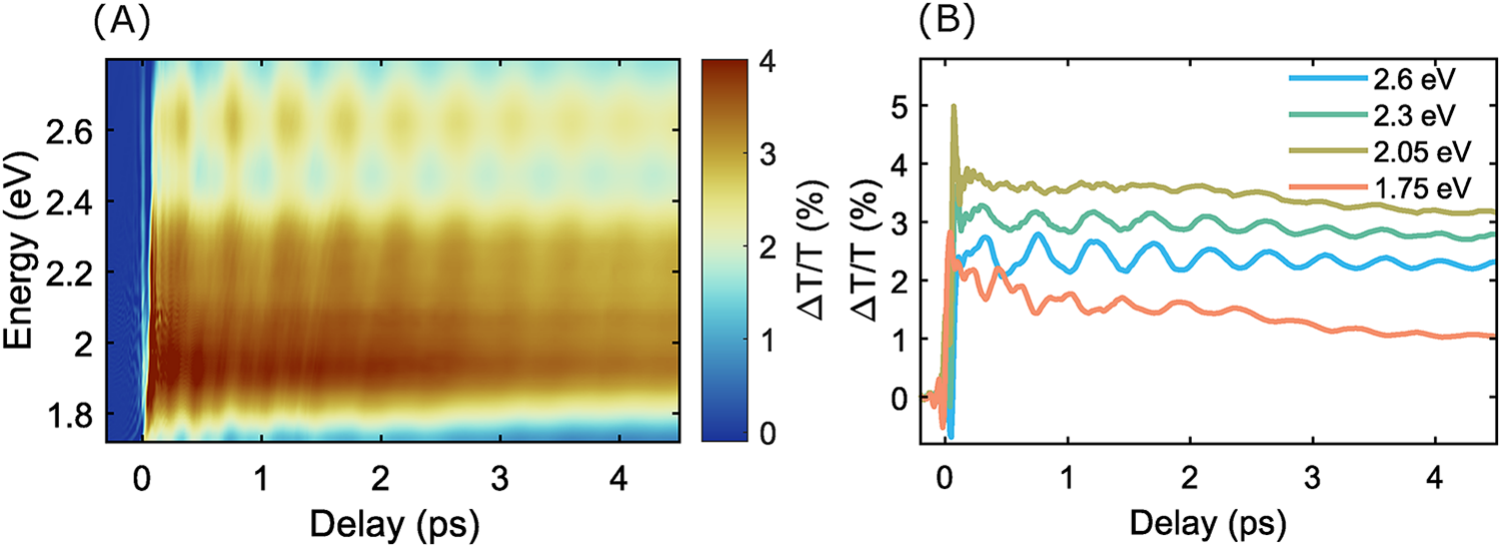
Transient transmissivity results. (A) Ultrafast transient transmissivity map of Bi_2_Se_3_ NPLs at 78 K following excitation with a 20 fs pump pulse centered at 2.1 eV. (B) Temporal evolution of the transient signal at selected probe energies, showing dynamics of coherent oscillations.

After the initial rise, oscillations emerge due to coupling between resonant electronic transitions and phonons launched by the pump pulse.^21^ The Raman-active phonon modes in Bi_2_Se_3_ are commonly observed at the following frequencies (see also Table S1 for a list of modes mentioned in the literature).

$$\begin{array}{ll} E_{g}^{\left(1\right)}:37\;{\mathrm{cm}}^{-1}\;\left(1.11\;\mathrm{THz}\right) & A_{1g}^{\left(1\right)}:73\;{\mathrm{cm}}^{-1}\;\left(2.19\;\mathrm{THz}\right)\\ E_{g}^{\left(2\right)}:132\;{\mathrm{cm}}^{-1}\;\left(3.96\;\mathrm{THz}\right) & A_{1g}^{\left(2\right)}:176\;{\mathrm{cm}}^{-1}\;\left(5.28\;\mathrm{THz}\right) \end{array}$$

E_g_ modes are shear modes, in which adjacent Bi and Se layers move parallel to each other as shown in Figure 1. A_1g_ modes are breathing modes, where adjacent atomic layers move perpendicularly to each other.^22,23^ Several studies consistently demonstrate the successful generation and detection of the fully symmetric A_1g_ phonon modes using ultrafast pump–probe spectroscopy.^38,47^ These modes are often observed alongside weaker E_g_ phonon modes, whose detection amplitudes are polarization dependent.^26,36,48–50^

To identify the modes launched in our NPLs, we isolated the oscillatory component of the transient signal by subtracting the electronic background, fitted using a biexponential function, and performing a Fast Fourier Transform (FFT) (see SI Figures S5–S9 for maps of the isolated electronic background and oscillations). Figure 4A presents the FFT spectra at selected probe energies. The spectrum is dominated by a peak at 2.12 THz (A_1g_^(1)^), corresponding to the ∼480 fs oscillation period visible in the time-domain traces (see Figure 3B). We also resolve the higher-frequency A_1g_^(2)^ mode at 4.95 THz, which is most prominent at the 1.75 eV probe edge. Both A_1*g*_ frequencies are red-shifted relative to literature values for bulk Bi_2_Se_3_, a softening attributed to phonon confinement effects in the ultrathin NPLs, possibly in combination with the red shift observed for surface phonons.^37,40,51,52^ In the low-frequency region, a mode appears at ∼0.4 THz. We assign this to the lowest-order confined acoustic breathing mode, as its frequency aligns well with the linear chain model for a slab of our measured thickness (see Supporting Text for detailed calculations).^22^ The in-plane E_g_ modes are largely suppressed, though a weak shoulder near 4.04 THz suggests a minor contribution from the E_g_^(2)^ mode.

**Figure 4:**
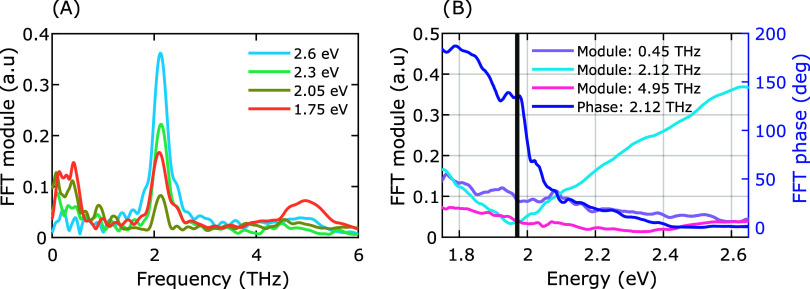
Fourier analysis of phonon modes. (A) Spectra of coherent oscillations as a function of oscillation frequency, obtained by taking the Fourier transform of the residual oscillatory part of the dynamics at selected probe energies. (B) Fourier transform module of oscillatory time domain data as a function of probe energy, for selected oscillation frequencies to show the probe energies at which each mode is active. The FFT phase (blue) at a frequency of 2.12 THz is also given as a function of probe energy. A phase shift of π is clearly seen at 1.97 eV, indicated by a black line. Measurements were performed at 78 K.

To determine the coupling pathways between specific lattice vibrations and electronic states, we analyzed the energy dependence of the phonon amplitude and phase, which is shown in Figure 4B. For the dominant A_1g_^(1)^ mode (2.12 THz), we observe a sharp minimum in oscillation amplitude at 1.97 eV, coinciding with a π phase jump.

This combination can be explained with the displacive excitation of coherent phonons (DECP)^41^ mechanism, where the coherent lattice distortion periodically modulates the energy of the electronic bands involved in an optical transition at 1.97 eV.^53^ As the phonon modulates the energy of the initial or final electronic state of this transition, the resulting shift in resonance energy causes the transient transmissivity signal to be opposite for probe photon energies slightly above and slightly below the transition center. This leads to out-of-phase oscillations (a π phase shift) on either side of the transition energy, while the FFT module reaches a minimum at the resonance peak. Hence, the A_1g_^(1)^ mode strongly couples to, and modulates, the electronic transitions near the absorption maximum, i.e., along the Γ–K line between VB1 and CB1/2. These states are not specifically associated with surface states.^14^ Although a similar minimum is observed around 2.35 eV for the 4.95 THz mode, the phase analysis of this mode is inconclusive due to a much lower signal-to-noise ratio (see Figure S8B for FFT maps of the complete pump–probe map). No clear minimum is observed for the 0.45 THz mode within our probe range.

To quantify the vibrational dephasing times at 293 and 78 K, we performed a global fit of the oscillatory components using three exponentially damped cosine functions, which is shown in Figure 5. The extracted frequencies are summarized in Table 1, and the energy dependent dephasing times (τ) of the three coherent phonon modes are given in Figure S10. The results show small differences between the two measurement temperatures. The optical modes show a thermal redshift at 293 K compared to 78 K. This softening is attributed to the thermal expansion of the lattice, which is expected to be most pronounced along the weakly bonded van der Waals *c*-axis.^54–56^ The thermal shift of the acoustic mode is very small, which is a result of its low initial frequency. Following the Grüneisen relation, the absolute shift of the mode scales with its initial frequency. Hence, the thermal softening of this low-energy interlayer mode is much smaller and barely detectable in our measurements. A calculation of the expected frequency shift is given in the Supporting Information.

**Figure 5:**
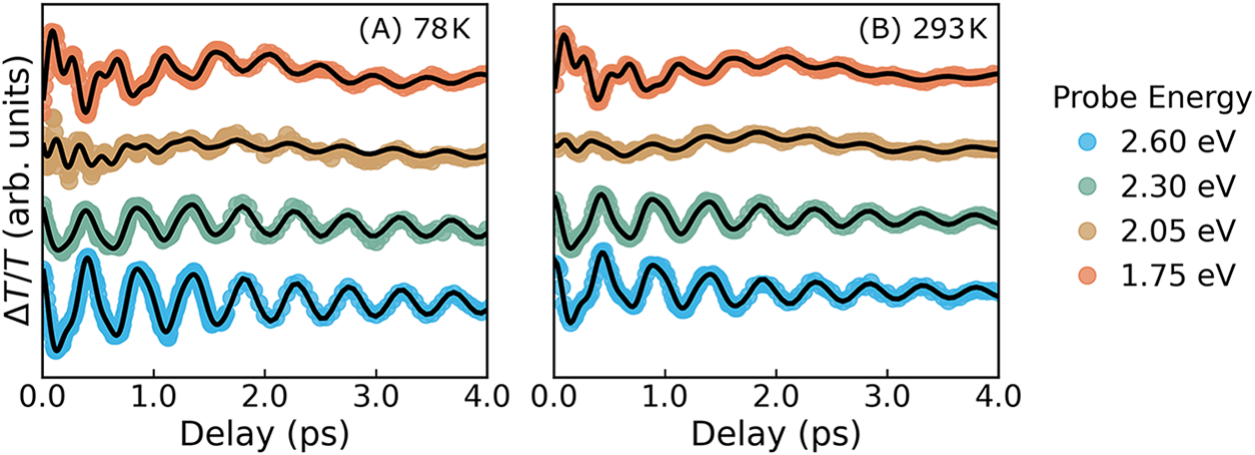
Time evolution of oscillations. Time evolution of the relative transmittance Δ*T*/*T* at probe energies of 1.75, 2.05, 2.3, and 2.6 eV. The time-evolution reflects the beating of the two out-of-plane A_1g_ modes and the breathing mode at 0.4 THz. The results are presented for measurements at 78 K (A) and 293 K (B). The fitting procedure is described in the Supporting Text.

**Table 1:** Extracted Phonon Frequencies from Figure 5, at 78 and 293 K. *f*_1_, *f*_2_ and *f*_3_ are the Acoustic Mode, A_1g_^1^ and A_1g_^2^, Respectively^a^

*T* (K)	*f*_1_ (**THz**)	*f*_2_ (**THz**)	*f*_3_ (**THz**)
293	0.380 ± 0.001	2.081 ± 0.001	4.880 ± 0.005
78	0.384 ± 0.001	2.125 ± 0.001	4.944 ± 0.004

a Errors represent the statistical uncertainty (±1σ) derived from the covariance matrix of the global least-squares fit, scaled by the reduced chi-squared.

The probe photon energy dependence of the phonon dephasing times is shown in the Supporting Information (see Figure S10). The trends in dephasing times at 78 and 293 K are quite similar, with most differences occurring around a probe energy of 2 eV, where the smallest oscillation amplitudes (see Figure S10C–D) are found. These small amplitudes prevent a reliable determination of the dephasing time in this energy range.

At both 78 and 293 K, the extracted dephasing time of the 2.1 THz (A_1g_^(1)^) mode appears to peak near 2 eV, aligning with the absorption maximum in Figure 2A. Note that the extracted τ at the peak reaches the 6 ps upper boundary constraint of the fitting model, and therefore does not represent the true dephasing time. The dephasing time drops to ∼2.5 ps at each side of the peak value for both temperatures. Converting this dephasing time to the frequency domain yields a spectral line width of Δν ≈ (πcτ)^−1^ ≈ 4.2 cm^–1^, which is in good agreement with Raman line widths reported for 1–7 QL films.^21–23^ The high-frequency A_1g_^(2)^ mode (∼4.9 THz) shows rapid dephasing (τ ∼ 500 fs), resulting in broad spectral features and limited observable oscillation cycles at both temperatures. Finally, the dephasing time of the acoustic breathing mode decreases at higher probe energies, i.e ∼3 ps at 1.7 eV compared to ∼0.5 ps at 2.4 eV. We tentatively attribute this decrease to phonon coupling in higher-lying electronic states, which typically have more relaxation pathways and higher scattering probabilities than states probed at lower energies.

Direct comparison of dephasing times with the literature is challenging due to their strong dependence on experimental parameters such as temperature, film thickness, and probe fluence. Nevertheless, our extracted values fall within the same order of magnitude as those previously reported for Bi_2_Se_3_.^24,31,50,57^

We will now address the absence of the Raman-active but nontotally symmetric E_g_ modes through a symmetry analysis using group theoretical arguments. In the Supporting Text, we have given a detailed description of the analysis, of which we will discuss the main results here.

Coherent phonons can be generated via DECP or impulsive stimulated Raman scattering (ISRS), for which Raman selection rules apply.^41,48^ ISRS involves nonresonant excitation through virtual states and can excite both symmetric and nonsymmetric phonon modes, and its efficiency is highly dependent on the polarization of the excitation pulse relative to the crystallographic axes of the material. In contrast, DECP is typically dominant under resonant excitation conditions, where ultrafast excitation perturbs the electron distribution and shifts the ionic equilibrium coordinates. Because our pump is resonant with a strong absorption feature between VB1 and CB1/2 at 1.97 eV in Bi_2_Se_3_, we attribute the coherent phonon generation to the DECP mechanism.

For electron–phonon coupling, general group-theory selection rules require that the direct product of the irreducible representations for the initial electronic state (Γ_*i*_), the phonon operator (Γ_ν_), and the final electronic state (Γ_*f*_) contains the totally symmetric representation A_1g_:

2 $$\Gamma_{f}⊗\Gamma_{\nu }⊗\Gamma_{i}⊇A_{1g}$$

In the DECP mechanism, the driving force arises from intraband deformation potentials, meaning the initial and final electronic states are identical (Γ_*i*_ = Γ_*f*_). Under this condition, the selection rule simplifies to [Γ_*i*_]^2^ ⊇ Γ_ν_. Hence, the symmetry of the excited phonon depends directly on the symmetry of the electronic conduction band states populated by the initial pump pulse. Our analysis of the Bi_2_Se_3_ band structure (see Figure S12 for symmetry analysis of the bands) indicates that electronic states at the high-symmetry points Γ and K exhibit well-defined A or E character. However, along the path between these points, the symmetry constraints are relaxed, and states may possess mixed A/E character. Spin–orbit coupling, which is not included here, may further relax these constraints.

We apply the selection rules derived above to the strongest transitions identified in our DFT calculations, namely those along the Γ–K line, from VB1 to the CB1/CB2 (see Figure 2 for *k*-resolved absorbance map and Figure S11 for calculated band structure). Along this path, the symmetry of the Bi_2_Se_3_ system reduces from *D*_3d_ to *C*_*s*_, where A_1g_ and E_g_ map to *A*′ and *A*′ + *A*″, respectively (Table S2). The symmetrized product table for *C*_*s*_ (Table S3) shows that

$$A^{\prime }⊗A^{\prime }=A^{\prime }\;\mathrm{and}\;A^{\prime \prime }⊗A^{\prime \prime }=A^{\prime }$$ 3

Hence, electronic states on the line Γ–K can couple to *A*′ phonons (related to A_1g_ phonons) via a single intraband electron–phonon interaction, but they cannot fully couple to E_g_-like phonons. This holds for all electronic states along Γ–K, regardless of the specific symmetry of the electronic states involved. A similar exclusion applies to the Σ segment (Γ–*M*).

Finally, the suppression of the E_g_ modes is enhanced by our experimental setup, as our isotropic detection is largely insensitive to the transient birefringence induced by shear modes, although the tilting of NPLs in the film might make detection possible.^41,48,58^ This combination of symmetry-restricted DECP generation and isotropic detection explains the experimental dominance of the A_1g_ modes and the absence of E_g_ contributions to the coherent oscillations.

## Conclusions

In conclusion, we have resolved the energy-dependent electron–phonon coupling pathways in 2D Bi_2_Se_3_ nanoplatelets using broadband ultrafast spectroscopy. Our measurements show the presence of out-of-plane A_1g_^(1)^ and A_1g_^(2)^ optical modes, alongside a low-frequency acoustic breathing mode that confirms the confinement of the lattice in the 2D limit. The Raman active E_g_ modes are absent. We find that the symmetry of the electronic states involved in the strongest transitions primarily permits coupling to the fully symmetric mode. In combination with our isotropic detection, this explains the dominance of these modes and the lack of E_g_ modes. Temperature-dependent studies between 78 K and room temperature show a softening of the A_1g_ frequencies, consistent with lattice anharmonicity and thermal expansion along the *c*-axis. By tracking the phonon amplitude across the visible spectrum, we identified that the A_1g_^(1)^ mode couples resonantly to electronic transitions near 1.97 eV. By using the band structure of Bi_2_Se_3_, we assign this resonance predominantly to inner-QL transitions between the VB1 and CB1/2 bands along the Γ–K high-symmetry line, coinciding with the system’s maximum linear absorbance. The knowledge of mode-specific coupling in 2D Bi_2_Se_3_ described here is important for understanding carrier cooling dynamics for optoelectronic and thermoelectric conversion.

## Supplementary Material



## Notes

**Supporting text:** Analysis of optical transitions via ***k***-resolved absorptance maps; theoretical calculations for the interlayer breathing mode frequency; estimation of the mode’s temperature dependence; data processing methodologies (incoherent background separation and global fitting); and symmetry analysis of forbidden/allowed phonon coupling. **Supporting tables:** A comparison of observed phonon modes from the literature (Table S1); correlation relations for group-subgroup pairs (Table S2); and symmetrized products of irreducible representations for D_3d_ and its subgroups (Tables S3–S4). **Supporting figures:** Pump and probe spectra (Figure S1); nanoplatelet structural characterization via TEM and EDX (Figure S2); cross-sectional TEM imaging of the film (Figure S3); ultrafast transient absorption maps and isolation of coherent oscillations at 293 and 78 K (Figures S4–S6); incoherent electronic lifetime fits (Figure S7); Fourier transform analysis (Figures S8–S9); extracted dephasing times and amplitudes from global fitting (Figure S10); theoretical ***k***-resolved absorptance maps (Figure S11); and band symmetry diagrams (Figure S12) (PDF)
