# Supplementary material for: Coherent Electron–Phonon Coupling in Two-Dimensional Bi2Se3 Nanoplatelets Studied with Ultrafast Spectroscopy
Source: J Phys Chem C Nanomater Interfaces. 2026 Apr 15;130(17):6213–20. doi: 10.1021/acs.jpcc.6c00732 (PMC13137260; doi:10.1021/acs.jpcc.6c00732)
Supplement: Supplementary file 1 [file jp6c00732_si_001.pdf]

Supporting information for:

Coherent electron-phonon coupling in two-dimensional Bi_2Se_3 nanoplatelets studied with ultrafast spectroscopy

Alessandro Baserga[1], Jara F. Vliem[2], Rhea Kumar[1], Riccardo Reho[2], Andrés Botello-Méndez[2], Daniel Vanmaekelbergh[2], Zeila Zanolli[2,*], Giulio Cerullo[1,*]

[1]Department of Physics, Politecnico di Milano, Piazza L. da Vinci 32, 20133 Milano, Italy
[2]Debye Institute for Nanomaterials Science, Utrecht University, Princetonplein 1, 3584 CC Utrecht, The Netherlands
[*]Email: z.zanolli@uu.nl, giulio.cerullo@polimi.it

The PDF file includes:

- **Supporting text:** Analysis of optical transitions via **k**-resolved absorptance maps; theoretical calculations for the interlayer breathing mode frequency; estimation of the mode's temperature dependence; data processing methodologies (incoherent background separation and global fitting); and a comprehensive symmetry analysis.
- **Supporting tables:** A comparison of observed phonon modes from the literature (Table S1); correlation relations for group-subgroup pairs (Table S2); and symmetrized products of irreducible representations for D_{3d} and its subgroups (Tables S3–S4).
- **Supporting figures:** pump and probe spectra (Figure S1); Nanoplatelet structural characterization via TEM and EDX (Figure S2); cross-sectional TEM imaging of the film (Figure S3); ultrafast transient absorption maps and isolation of coherent oscillations at 293 K and 78 K (Figures S4–S6); incoherent electronic lifetime fits (Figure S7); Fourier transform analysis (Figures S8–S9); extracted dephasing times and amplitudes from global fitting (Figure S10); theoretical **k**-resolved absorptance maps (Figure S11); and band symmetry diagrams (Figure S12).

Supporting text

Analysis of transitions through pump excitation

As described for 6 QL Bi_2Se_3[1], the strongest transitions within a certain energy range can be found from **k**-resolved absorptance plots. Such plots depict the integrated theoretical absorptance (color scale, arbitrary units) within a chosen energy range, projected onto the (E=0, k_x, k_y) plane (2D Brillouin zone). We will use the computed data for 6 QL Bi_2Se_3 here as well, since it will be very similar to the 4 QL case as the band gap computed with DFT-GW only differs by 58 meV(4 QL)−27 meV(6 QL)=31 meV[2]. For the excitation energy of 1.90-2.30 eV used in our experiment, the corresponding theoretical excitation is 1.65-2.05 eV, which takes into account a shift between theory and experiment. From the **k**-resolved absorptance maps in this energy range, see Figure S11, we find that the strongest absorptance occurs between Γ–K and on the line Γ–M. These transitions are identified to occur between the upper VB and CB1/CB2, as shown in the band structure of Figure 2C. The arrow on the line Γ–M shows only one out of many possible transitions on this line.

Calculating interlayer breathing mode frequency

The frequency ω of the breathing mode is given by[3, 4]:

$$\omega_\alpha = \sqrt{\frac{K_z}{2\mu\pi^2c^2}\left(1-\cos\left(\frac{(\alpha-1)\pi}{N}\right)\right)} \tag{S1}$$

where N is the number of quintuple layers (QLs), K the interlayer force constant, α the mode index, μ the mass of a single QL per square meter, and c the speed of light in vacuum. Experimentally determined constants for Bi_2Se_3 were used ($K_z = 5.26 \times 10^{19}$ N m^{-3}, $\mu = 7.5 \times 10^{-6}$ kg m^{-2}, $\alpha = 2$). Based on our TEM analysis, the nanoplatelet thickness follows a distribution of 4.3 ± 1.1 QLs. We calculated the breathing mode frequencies for the lower and upper bounds of this range. For the thickest platelets ($N = 5.4$), the model predicts a frequency of 8.1 cm^{-1} (0.24 THz), while for the thinnest platelets ($N = 3.2$), the frequency increases to 13.2 cm^{-1} (0.40 THz). The experimental frequency of the acoustic phonon mode falls between these boundaries.

Temperature dependence of breathing mode

We estimate the change in crystal volume as a function of temperature as:

$$\frac{\Delta V}{V} \approx (2\frac{\Delta a}{a} + \frac{\Delta c}{c}) \approx (2\alpha_\perp + \alpha_\parallel)\Delta T$$

where, from ref.[5], $\alpha_\perp = 1.1 \times 10^{-5}$ K^{-1}, and $\alpha_\parallel = 1.9 \times 10^{-5}$ K^{-1}. In our experiment, $\Delta T = -220K$. This yields a fractional change of $\Delta V/V \approx -0.00902$. We have used the Grüneisen relation to calculate the resulting change in the phonon frequency:

$$\frac{\Delta\omega}{\omega_0} = -\gamma\frac{\Delta V}{V}$$

where V is the volume of the material, γ is the Grüneisen parameter, and ω_0 is the initial frequency of the phonon mode. Using a Grüneisen parameter of $\gamma = 1.4$, obtained from ref. [5], and the acoustic mode frequency at 298 K of $\omega_0 = 12.68$ cm^{-1}, we calculate a frequency shift of $\Delta\omega \approx 0.16$ cm^{-1}.

Hence, the predicted frequency at 78 K is 12.84 cm^{-1}. This corresponds to approximately 0.385 THz, which is in agreement with our measured value of 0.384 THz at 78 K. Thus, while the acoustic mode is indeed temperature-dependent, the absolute magnitude of its thermal shift is small due to the mode's low initial frequency.

Data processing

Separation of incoherent electronic background and coherent phonon oscillation from the transient absorption maps

The transient absorption map comprises two distinct contributions: a coherent oscillatory component driven by lattice phonons and an incoherent exponential decay reflecting electronic population relaxation. To isolate the electronic dynamics from the overall signal, we fitted the transient trace at each probe wavelength to a biexponential decay model with a constant offset:

$$I(t) = A_1 e^{-t/\tau_1} + A_2 e^{-t/\tau_2} + y_0 \tag{S2}$$

The fitting window for each trace was initialized immediately following the pump-induced signal rise, defined mathematically as the maximum of the numerical time derivative of the signal. The separated electronic and phononic contributions are plotted in Figures S4 and S5.

To obtain a more precise quantitative estimate of the incoherent electronic relaxation lifetimes, we extracted the transient dynamics at the probe wavelength exhibiting the maximum signal amplitude at both 293 K and 78 K. These kinetic traces were fitted using a tri-exponential decay model analytically convoluted with a Gaussian instrumental response function (IRF). The fitting function is expressed as:

$$I(t) = \sum_{i=1}^{3} \frac{A_i}{2} \exp\left(\frac{\sigma^2}{2\tau_i^2} - \frac{t-t_0}{\tau_i}\right) \left[1 + \text{erf}\left(\frac{t - t_0 - \sigma^2/\tau_i}{\sqrt{2}\sigma}\right)\right] \tag{S3}$$

where A_i and τ_i represent the amplitude and lifetime of the i-th decay component, t_0 is the temporal zero-delay (time overlap), σ defines the width of the Gaussian IRF, and erf denotes the error function. The extracted kinetic traces, alongside their corresponding fits and residuals, are presented in Figure S7.

From this analysis, we extract an intermediate relaxation lifetime (τ_2) of 3.91 ps at 293 K, which increases to 7.65 ps at 78 K. The fastest decay component ($\tau_1 \approx 20$ fs) approaches the pump pulse duration and is included primarily to account for coherent artifacts occurring during pulse overlap. Conversely, the longest decay constant ($\tau_3 > 15$ ps) exceeds the measured temporal window, capturing slower, long-lived recombination processes.

Global fitting

To extract the dynamics of the coherent phonons, the transient data $\Delta T/T(t, E)$ up to 4.5 ps was analyzed using a non-linear least-squares global fitting in Python (SciPy). The 4.5 ps boundary was chosen as the oscillations were only well resolved up to this point, see also Figure S7 for the full signal up to 25 ps. The oscillations were modeled as a superposition of three underdamped oscillators superimposed on a linear slowly varying background. The global fit model is defined as:

$$\frac{\Delta T}{T}(t, E) = \sum_{i=1}^{3} A_i(E) e^{-t/\tau_i(E)} \cos(2\pi f_i t + \phi_i(E)) + B(E) + C(E)t \tag{S4}$$

where A_i, τ_i, f_i, and ϕ_i are the amplitude, dephasing time, frequency (in THz), and initial phase of the i-th phonon mode, respectively, and t is the pump-probe delay time (in ps). $B(E)$ and $C(E)$ represent the offset and slope of the non-oscillatory electronic background. The phonon frequencies f_i were treated as global parameters (shared across all probe energies E), while the amplitudes, phases, dephasing times, and background parameters were treated as local variables dependent on the probe energy.

To determine the uncertainty (σ) of each parameter, we calculated a covariance matrix using the pseudo-inverse of the Jacobian at the optimum. These values were then scaled by the residuals' reduced chi-squared to provide more realistic error estimates. In Figure S10, the

energy-dependent dephasing times and the amplitudes are presented with their 95% confidence intervals ($\pm 2\sigma$).

Symmetry analysis

Initial considerations:

- The probability for optical transitions is largest at critical points of the joint density of states. These points are often related to critical points (minima, maxima, saddle points) of the band structure.
- Critical points often appear at high symmetry points or high symmetry lines.
- In Bi_2Se_3, the absorption is dominated by transitions along the $\Gamma \to K$ line (called Λ) and the $\Gamma \to M$ line (called Σ).
- Bi_2Se_3 crystal structure belongs to the space group R$\bar{3}$m (D_{3d} point group).
- The BZ zone center point ($\mathbf{\Gamma}$ has the same point group as the crystal (D_{3d})), while the Λ and Σ lines have point groups C_s and C_2, respectively.
- Our analysis focuses on $\mathbf{\Gamma}$, Λ and Σ for the electronic states
- We assume that the pump and probe beams are aligned, meaning that we will not probe transitions were momentum transfer has occurred. For this reason, we consider only $\mathbf{\Gamma}$ phonons.

Introduction to general group theory

Physical processes, such as optical excitation, induce transitions between the eigenstates of a system. The quantum mechanical description of these processes typically involves evaluating matrix elements of the form $\langle\psi_f|O|\psi_i\rangle$, where ψ_i and ψ_f represent the initial and final states of the system, respectively, and O is the operator corresponding to the physical process. This matrix element is an integral performed over all space. For this integral to be non-zero, the integrand must contain a component that transforms as the totally symmetric representation of the point group of the system.

Instead of directly evaluating the complex mathematical expression for the integral, we can use group theory to determine if the matrix element is non-zero. The condition for a non-zero matrix element is that the direct product of the irreducible representations (irreps) of the final state (Γ_f), the operator (Γ_O), and the initial state (Γ_i) must contain the totally symmetric irreducible representation, denoted as A_{1g}:

$$\Gamma_f \times \Gamma_O \times \Gamma_i \supseteq A_{1g} \tag{S5}$$

If the decomposition of this triple direct product contains A_{1g}, the matrix element is non-zero, and the transition is considered allowed. Conversely, if the triple direct product does not contain A_{1g}, the matrix element is zero because the integrand will be odd, resulting in a forbidden transition. Allowed transitions generally show up as strong signals in the spectra of a material, while forbidden transitions lead to much weaker signals.
Since the direct product of any irrep with itself will always contain the totally symmetric representation in its decomposition, we can use equivalent simpler forms, which state that the direct product of any two representations must contain the third, such as $\Gamma_i \otimes \Gamma_O \supseteq \Gamma_i$ or $\Gamma_f \otimes \Gamma_f \supseteq \Gamma_O$.

Application to transient absorption

For an initial optical transition to be allowed, we have the condition $\Gamma^*_{elec} \otimes \Gamma_{O_{opt}} \otimes \Gamma_{elec} \supseteq A_{1g}$, where Γ_{elec} and Γ^*_{elec} represent the irreps of the initial and final electronic states involved in the optical transition, and $\Gamma_{O_{opt}}$ is the representation of the optical operator (typically a dipole operator, transforming as x, y, or z).

Following the initial excitation, we must examine the coupling of coherent phonons. In a transient absorption experiment, a pump pulse excites the crystal, generating coherent phonons via ISRS or DECP. A subsequent probe pulse then characterizes the changes in the optical properties due to these phonons. We are primarily interested in the selection rules for the generation of these coherent phonons by the pump pulse, although the coupling to the probe pulse follows similar symmetry considerations.

The nature of the operator responsible for coherent phonon generation depends on the specific excitation mechanism. Since our pump pulse is resonant with a strong electronic transition at 1.97 eV (see Figure 4B), the dynamics are expected to be DECP-dominated. The pump pulse rapidly excites the electronic system, leading to a change in the electronic potential energy surface. This change results in a shift of the equilibrium positions of the atoms, thus driving specific phonon modes. The atoms then oscillate coherently around these new equilibrium positions. In this DECP scenario, the effective interaction Hamiltonian, H_{e-ph}, describes the coupling between electrons and phonons. Hence, the transition probability for a process where an electron in an initial state $|i\rangle$ emits a phonon ν and transitions to a final state $|f\rangle$ is proportional to the matrix element $\langle f, \nu | H_{e-ph} | i, 0\rangle$. Considering the symmetry of the operator, the part that is linear in the phonon coordinate q_ν has the same symmetry as the phonon mode ν, i.e., Γ_ν. This means that we can write our condition for an allowed transition as:

$$\begin{aligned} &\Gamma_f \otimes \Gamma_\nu \otimes \Gamma_i \supseteq A_{1g} \\ &\text{or} \\ &\Gamma_i \otimes \Gamma_f \supseteq \Gamma_\nu \end{aligned} \tag{S6}$$

For intraband transitions, where the initial and final electronic states belong to the same band and have the same symmetry at a given k-point ($\Gamma_i = \Gamma_f$), this condition simplifies to $[\Gamma_i]^2 \supseteq \Gamma_\nu$, meaning the symmetrized square of the electronic state's representation must contain the phonon's representation. Note that the simplified condition is also true if the transition is interband, rather than intraband, as long as the irrep of the initial and final electronic states is the same. This is often the case for few-layer Bi_2Se_3, where band manifolds correspond to electronic states located at different layers, and thus have the same symmetry but different energy due to Pauli principle.

The case of Bi_2Se_3

Our system of interest, layered Bi_2Se_3, has a crystal structure belonging to the space group R$\bar{3}$m (D_{3d} point group). With 5 atoms in the unit cell, the 15 vibrational degrees of freedom give rise to 15 phonon branches. At the zone center (Γ point), these modes decompose into the irreps: $\Gamma_{vib} = 2E_g + 2A_{1g} + 2E_u + 2A_{1u}$. Since photons have very little momentum, optical spectroscopy like Raman and pump-probe primarily probes zone-center phonons. For coupling of coherent phonons excited by pump/probe experiments via an intraband displacive mechanism, the symmetry of the excited phonon mode Γ_ν must be contained in the symmetrized direct product of the irrep of the electronic states involved in the excitation process, i.e. $[\Gamma_i]^2 \supseteq \Gamma_\nu$. For this analysis, we must first determine the symmetry of our system at the **k**-point of interest in the Brillouin zone. At the center of the Brillouin zone, the point group defining the irreps of the electronic states is the same as that of the crystal, D_{3d}. Away from Γ, the system's symmetry is reduced and is described by a so-called little group that contains fewer symmetry

elements than the full point group. The correlation table, Table S2, can be used to related the symmetry of the full system to the lower-symmetry subgroup. When the symmetry at the **k**-point of interest is known, we can use tables that show the symmetrized products of irreps, for example Tables S3 and S4, to determine whether coupling is allowed.

We analyzed the band structure of 6 QL Bi_2Se_3, without spin-orbit coupling to obtain the irreps of the **k**-points at Γ and K as shown in Figure S12. Between the high symmetry points of the Brillouin zone, the symmetry constraints found for Γ and K are relaxed, and states may have A/E mixed character. Spin–orbit coupling, not included here, may further relax symmetry constraints.

Application to 2D Bi_2Se_3

We will first detail the calculation for 2D Bi_2Se_3 at Γ, although this calculation can be generalized to any other **k**-point. At the center of the Brillouin zone, the point group defining the irreps of the electronic states is the same as that of the crystal, D_{3d}. Looking at the symmetrized products table of D_{3d} (Table S4), we see that the symmetrized squares of E_g and E_u contain A_{1g} and E_g. This implies that if the pump pulse creates electronic excitations into states with E_g or E_u symmetry, these electronic states can couple to both A_{1g} and E_g phonons, via intraband (or same symmetry) electron-phonon coupling. In contrast, the symmetrized squares of A_{1g}, A_{1u}, A_{2g}, and A_{2u} only contain A_{1g}, suggesting that electronic states with these symmetries can only couple to A_{1g} phonons through intraband processes. With our symmetry analysis of the band structure of Bi_2Se_3 (Figure S12), we can examine which states are excited in an experiment to check whether their symmetry allows electron-phonon coupling. Around Γ, the states in Figure S12 exhibit well-defined A or E character. Hence, at Γ, both A and E-type phonons are allowed to couple depending on the targeted bands.

We will now discuss the selection rules at the K-point. Considering the little group corresponding to the K point in the Brillouin zone, the symmetry is C_{3v}. To relate the representations for the Γ phonons in D_{3d} to the representations in C_{3v} that describe the electronic states, we use the correlation relations (Table S2). We observe that the E_g mode in D_{3d} correlates to the E mode in C_{3v}. The symmetrized products table for C_{3v} (Table S3) shows that the symmetrized square of the E representation contains A_1 and E. Since A_{1g} in D_{3d} correlates to A_1 in C_{3v}, this indicates that E electronic states at the K point can couple to both A_1 (related to A_{1g}) and E (related to E_g) phonons. However, A_1 excitations can only couple to A_{1g} phonons.

Based on this analysis, which considers the displacive excitation of coherent phonons and intraband electron-phonon coupling, we generally expect phonons of A_{1g} symmetry to be strongly excited, as they can couple to electronic states of various symmetries across the Brillouin zone, regardless of excitation energy. The excitation of E_g phonons is expected to be weaker (see also main text for analysis along Σ and Λ).

Supporting tables

Table S1: Table of various observed phonon modes in Bi_2Se_3, and the reference in which the mode was described. Bulk means samples that show 3D TI behaviour, i.e. samples thicker than 7 QLs.

**Mode**	**Sample type**	$\tilde{\nu}$ **(cm^{-1})**	**Freq. (THz)**	**Ref.**
Acoustic	Bulk single crystals	1.1	0.033	1
Acoustic	Films of 2 QLs on Al_2O_3	5.0	0.15	7
Acoustic	Films of 5 QLs on Al_2O_3	13.7	0.41	7
$E_g^{(1)}$	NPLs of ≥4 QLs	37.0	1.11	2
$E_g^{(1)}$	Bulk single crystals	37.0	1.11	3
$E_g^{(1)}$	Bulk films on Al_2O_3 and single crystals	39.0	1.17	5
$E_g^{(1)}$	Bulk films on BaF_2	36.7	1.1	6
$E_g^{(2)}$	NPLs of ≥4 QLs	131.0	3.93	2
$E_g^{(2)}$	Bulk single crystals	131.0	3.93	3
$E_g^{(2)}$	Bulk films on Al_2O_3 and single crystals	137.0	4.11	5
$E_g^{(2)}$	Bulk films on BaF_2	135.1	4.05	6
$E_g^{(2)}$	Bulk films on SiO_2	128.3	3.85	8
$A_{1g}^{(1)}$	Bulk single crystals	72.38	2.17	1
$A_{1g}^{(1)}$	NPLs of ≥4 QLs	72.0	2.16	2
$A_{1g}^{(1)}$	Bulk single crystals	72.0	2.16	3
$A_{1g}^{(1)}$	Bulk single crystals	74	2.23	4
$A_{1g}^{(1)}$	Bulk films on Al_2O_3 and single crystals	75	2.25	5
$A_{1g}^{(1)}$	Bulk films on BaF_2	75	2.25	6
$A_{1g}^{(1)}$	Bulk films on SiO_2	70.8	2.12	8
$A_{1g}^{(2)}$	NPLs of ≥4 QLss	174.0	5.22	2
$A_{1g}^{(2)}$	Bulk single crystals	175.0	5.25	3
$A_{1g}^{(2)}$	Bulk films on Al_2O_3 and single crystals	180.0	5.40	5
$A_{1g}^{(2)}$	Bulk films on SiO_2	174.0	5.22	8

1. Kumar, N. *et al.* Phys. Rev. B Condens Matter Mater Phys 83.23 (2011): 235306.
2. Zhang, J. *et al.* Nano Lett. 11.6 (2011): 2407-2414.
3. Irfan, B. *et al.* J. Appl. Phys. 115.17 (2014): 173506
4. Sobota, J. A. *et al.* Phys. Rev. Lett. 113.15 (2014): 157401.
5. Kung, H.H. *et al.* Phys. Rev. B 95.24 (2017): 245406.
6. Melnikov, A. A. *et al.* Phys. Rev. B 97.21 (2018): 214304.
7. Glinka, Y. D. *et al.* Sci. Rep. 12 (2022).
8. Saini, S. K. *et al.* Adv. Mater. Interfaces 10.3 (2023): 2201650.

Table S2: Correlation relations for several group-subgroup pairs in Bi_2Se_3.

$D_{3d}(\bar{3}m)$	C_{3v} $(3m)$	C_s (m)	C_2 (2)
A_{1g}	A_1	A'	A
A_{1u}	A_2	A''	A
A_{2g}	A_2	A''	B
A_{2u}	A_1	A'	B
E_g	E	$A' + A''$	$A + B$
E_u	E	$A' + A''$	$A + B$

Table S3: Symmetrized Products of irreducible representations for C_{3v}(3m), C_s(m), and C_2(2).

	C_{3v}(3m)			C_s(m)		C_2(2)	
	A_1	A_2	E	A'	A''	A	B
$[A_1 \otimes A_1]$	1	·	·				
$[A_2 \otimes A_2]$	1	·	·				
$[E \otimes E]$	1	·	1				
$[A' \otimes A']$				1	·		
$[A'' \otimes A'']$				1	·		
$[A \otimes A]$						1	·
$[B \otimes B]$						1	·

Table S4: Symmetrized products of irreducible representations of D_{3d}.

$D_{3d}(\bar{3}m)$	A_{1g}	A_{1u}	A_{2g}	A_{2u}	E_u	E_g
$[A_{1g} \otimes A_{1g}]$	1	·	·	·	·	·
$[A_{1u} \otimes A_{1u}]$	1	·	·	·	·	·
$[A_{2g} \otimes A_{2g}]$	1	·	·	·	·	·
$[A_{2u} \otimes A_{2u}]$	1	·	·	·	·	·
$[E_u \otimes E_u]$	1	·	·	·	·	1
$[E_g \otimes E_g]$	1	·	·	·	·	1

Supporting figures

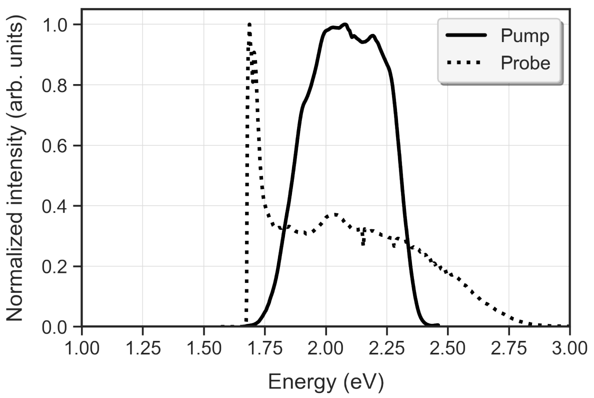

Figure S1: **Pump and probe spectra.**
Normalized pump and probe spectra used in the experiments.

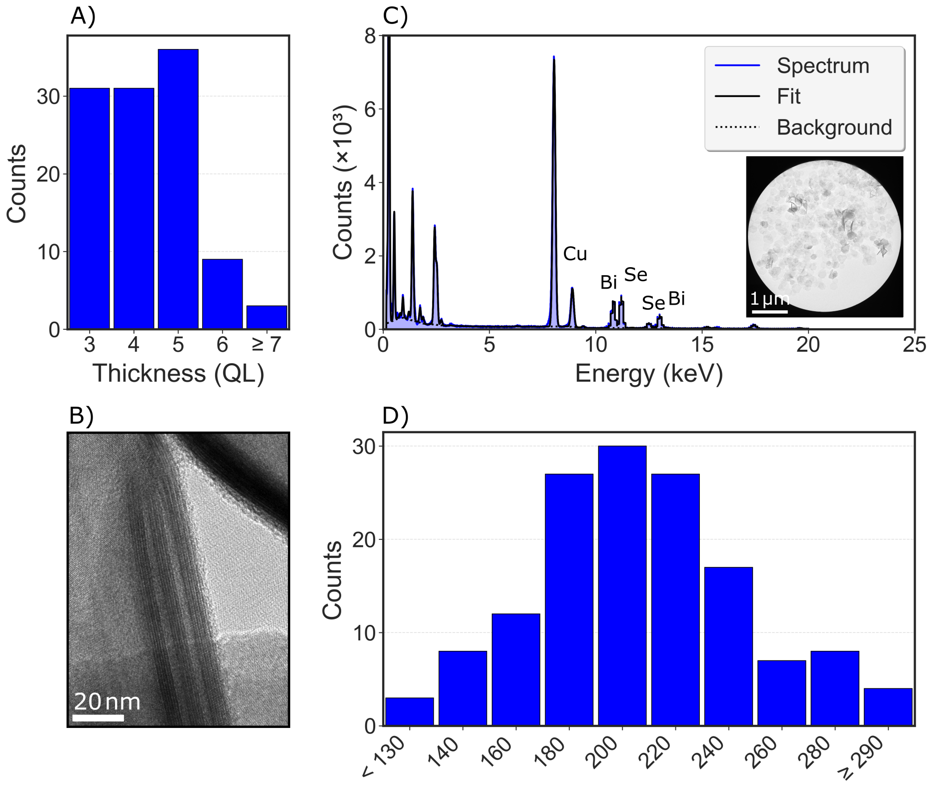

Figure S2: **Characterization of nanoplatelets.**
A) Thickness of 110 nanoplatelets, obtained by counting quintuple layers of edge-on platelets in TEM (see TEM image in B). C) EDX measurement of the area in the inset, from which a composition of 37:63 Bi:Se atomic percentage was obtained. D) Maximal diameter of 140 platelets, obtained from TEM. The bin average is given, for a bin width of 20 nm.

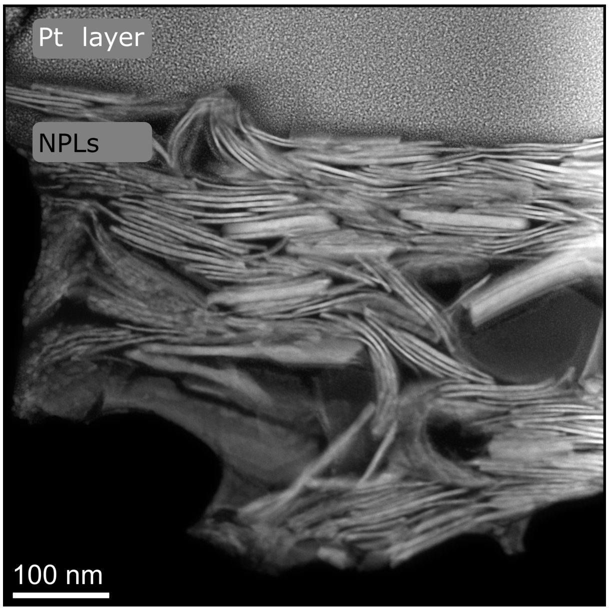

Figure S3: **Cross-sectional TEM image of Bi_2Se_3 NPL film.**
The image shows a cross section of a drop-cast NPL film, in which stacked NPLs are visible from the side. Most NPLs tend to lie relatively flat on the substrate when drop-cast, although often at a slight angle compared to the substrate. The top dark region corresponds to the protective Pt cap used during the milling process.

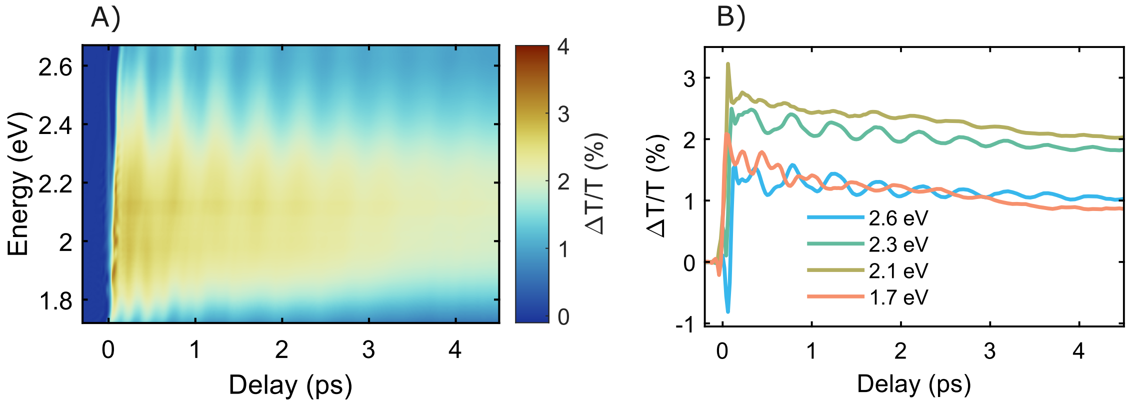

Figure S4: **Additional data at** 293 K**.**
A) Ultrafast transient absorption map of Bi_2Se_3 platelets at 293 K following excitation with a 20 fs pump pulse centered at 2.1 eV. B) Temporal evolution of the transient signal at selected probe energies showing dynamics of coherent oscillations.

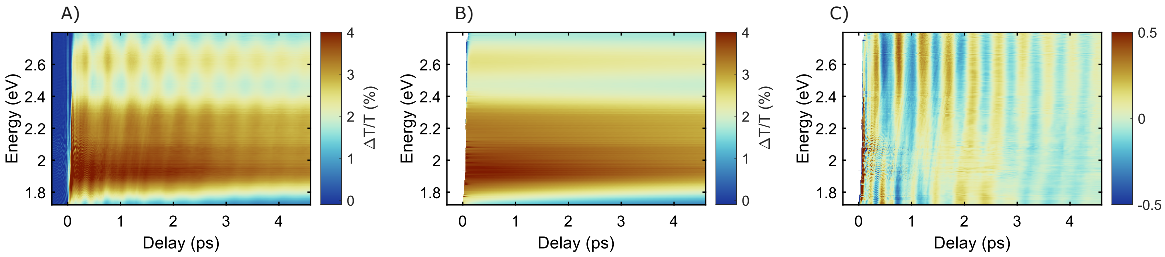

Figure S5: **Isolating phonon modes at 78 K.**
A) Ultrafast transient absorption map of Bi_2Se_3 platelets at 78 K following excitation with a 20 fs pump pulse centered at 2.1 eV. B) Ultrafast transient absorption map of the incoherent contribution. C) Ultrafast transient absorption map of the coherent oscillations. To separate the contribution of band population from the coherent oscillations, we fitted a bi-exponential decay function with a constant background to account for the incoherent signal given by the electronic population. The result of such a fit is shown in B), and by subtracting it from the raw signal we can isolate coherent oscillations C). We assign the oscillatory part of the signal presented in C) to phonon populations of different modes.

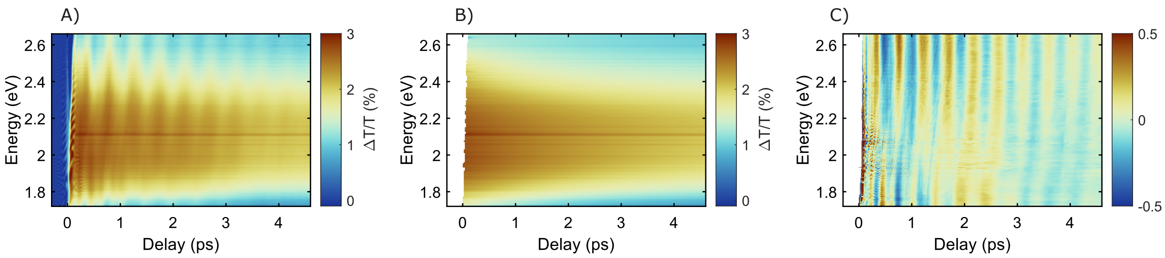

Figure S6: **Isolating phonon modes at 293 K.**
A) Ultrafast transient absorption map of Bi_2Se_3 platelets at 293 K following excitation with a 20 fs pump pulse centered at 2.1 eV. B) Ultrafast transient absorption map of the incoherent contribution. C) Ultrafast transient absorption map of the coherent oscillations. The same procedure for isolating the modulations was used as for Figure S5.

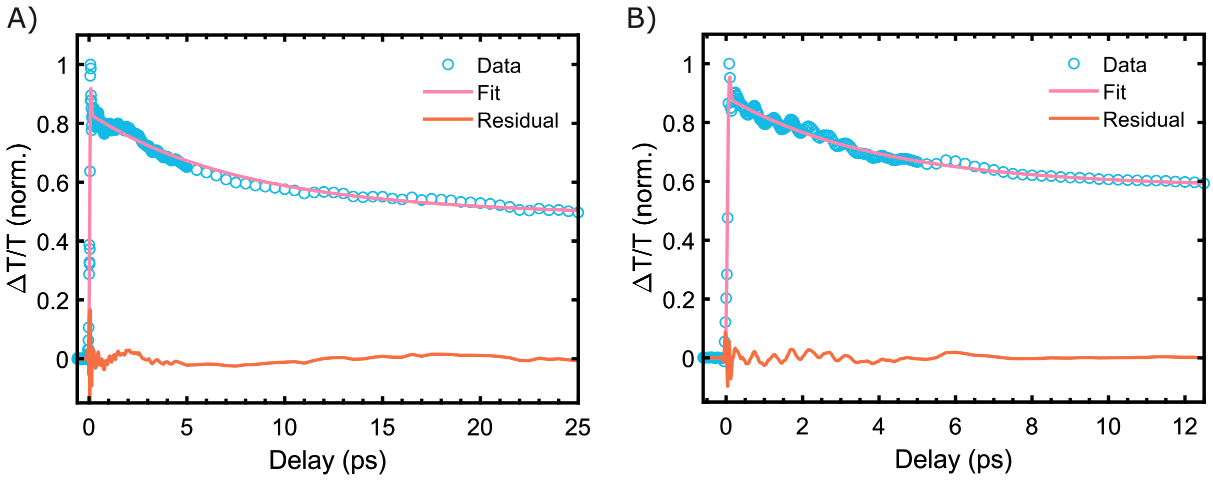

Figure S7: **Fits of the incoherent electronic lifetimes at 78 and 293 K.**
A) Dynamics extracted at the maximum of the bleach signal (641 nm) at 78 K fitted with a triexponential decay convolved with the IRF of the setup. Residual oscillations are shown below. A) Dynamics extracted at the maximum of the bleach signal (591 nm) at 293 K fitted with a triexponential decay convolved with the IRF of the setup. Residual oscillations are shown below.

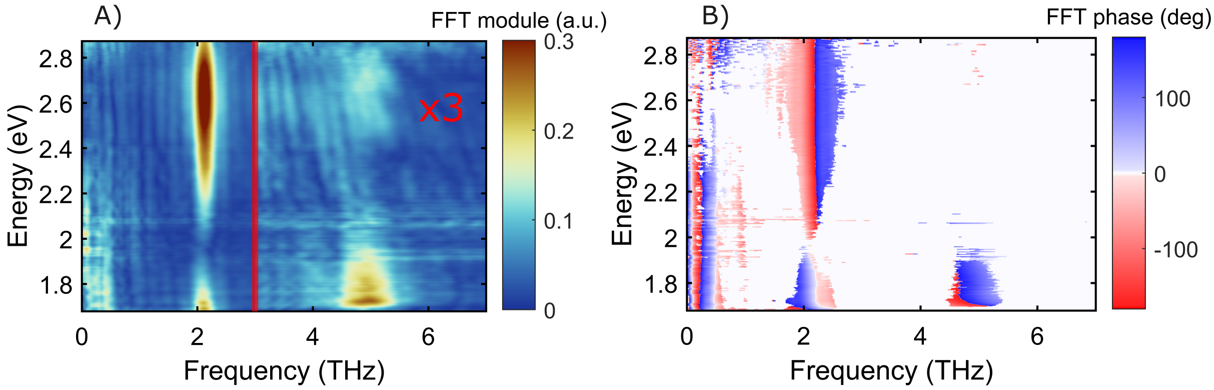

Figure S8: **Fourier analysis at 78 K.**
A) Absolute value of the Fourier transform of the oscillatory component of the data taken at 78 K. Data points above 3 THz are multiplied by 3. B) Phase of the Fourier transform of the oscillatory component of the data taken at 78 K. The oscillation signal shown in Figure S5C is Fourier transformed to extract information regarding the different modes observed. Before applying the Fast Fourier Transform algorithm to the data, we linearly interpolated the data to the smallest sampling step present at each dynamic. In the calculation of the phase, a threshold is applied to account for rounding off errors. Moreover, a median filter in time and energy, single value denoising and zero padding are applied. Figures S8A,B show false color plots of the amplitude and phase of the oscillations in a frame with the probe photon energy on the Y axis and the phonon frequency (0–7 THz) on the X-axis. This procedure is repeated for the data taken at 293 K in Figure S6C as shown in Figure S9.

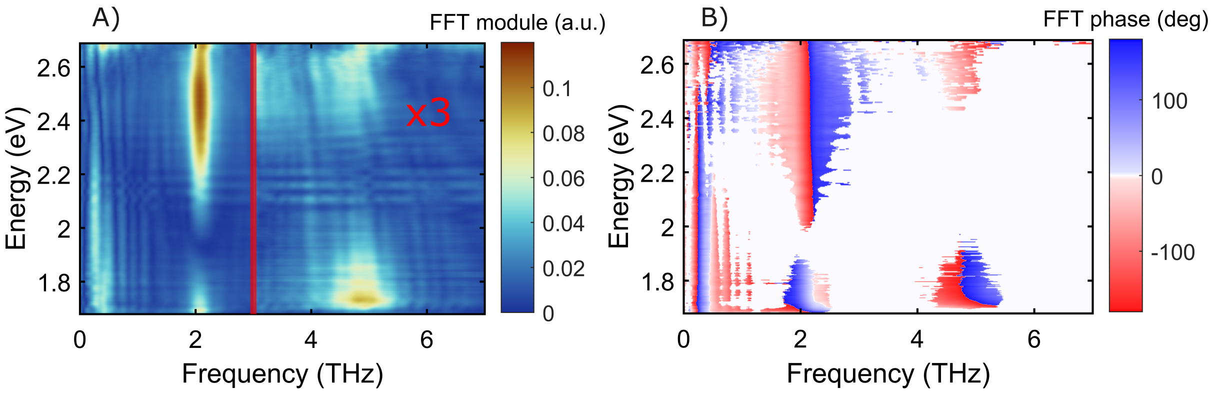

Figure S9: **Fourier analysis at 293 K.**
A) Absolute value of the Fourier transform of oscillatory component of the data taken at 293 K. Data points above 3 THz are multiplied by 3. B) Phase of the Fourier transform of the oscillatory component of the data taken at 293 K. Procedure described in Figure S8.

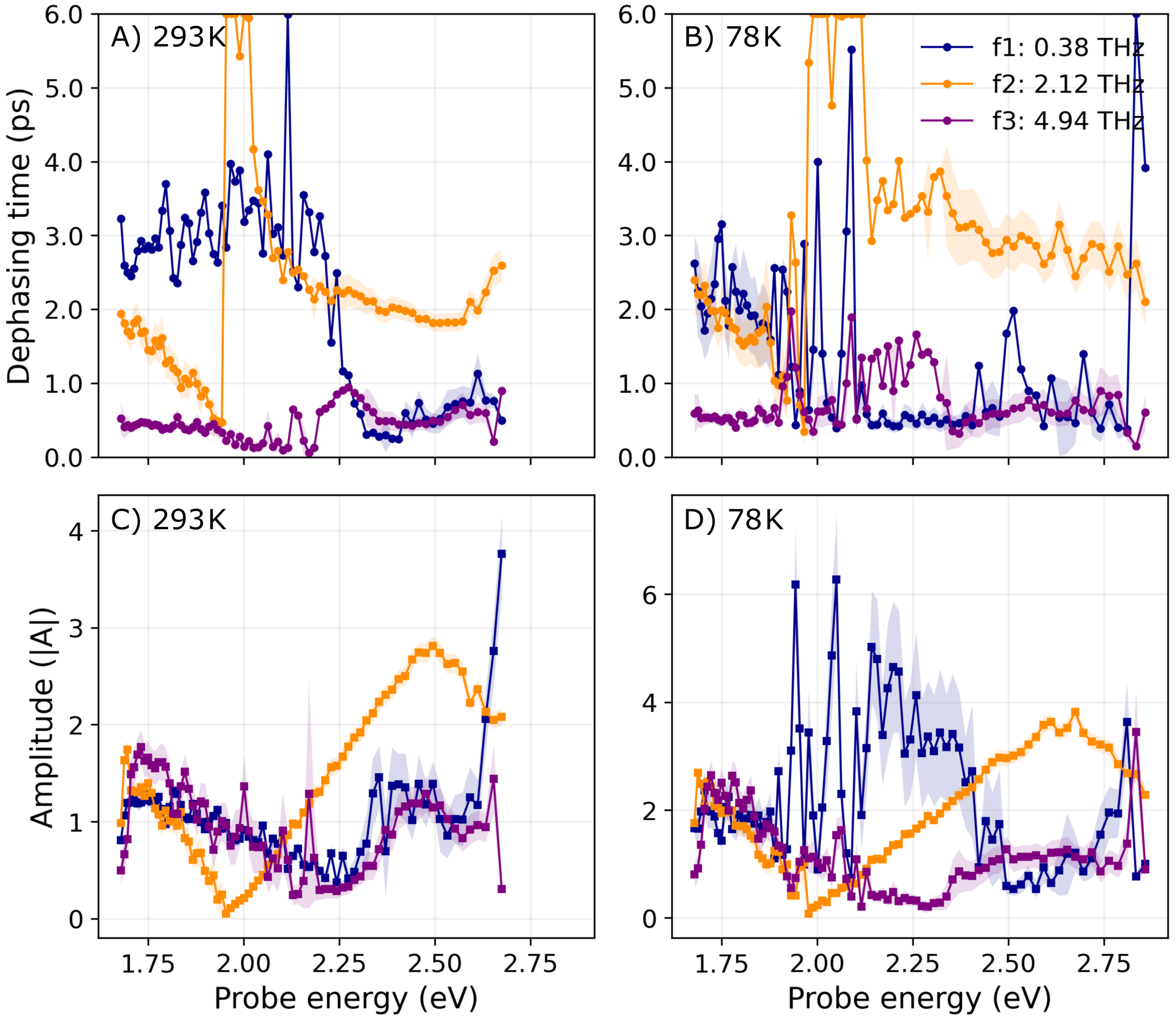

Figure S10: **Dephasing time τ (A,B) and absolute oscillatory amplitude $|A|$ (C,D) as function of probe energy for global parameters f_1, f_2, and f_3, at 293,K and 78,K.** The plotted data represents the output from the global least-squares fit. Shaded regions represent the 95% confidence intervals (2σ) derived from the fit covariance. Note that a maximum lifetime value of 6 ps was imposed. Around 2 eV, the amplitude is very small and the fit is therefore not reliable, explaining the maximum lifetimes.

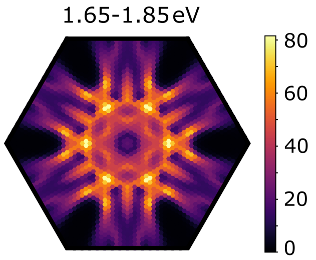

Figure S11: **k-resolved absorptance map integrated between 1.65-**2.05 eV **for 6 QL Bi_2Se_3.** The color bar represents the strength of the calculated absorbance in arbitrary units.

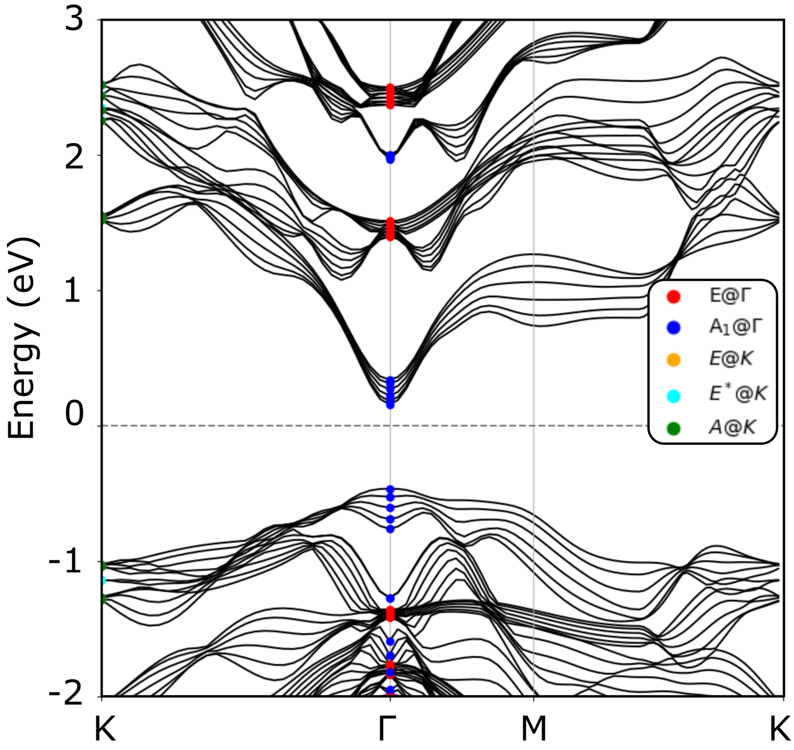

Figure S12: **Symmetry of bands in 6 QL Bi_2Se_3.**
Band structure of 6 QL Bi_2Se_3, without spin-orbit coupling, showing the symmetry of the **k**-points at Γ and K.

References

[1] J. F. Vliem, S. A. Cayan, R. Reho, A. R. Botello-Méndez, P. Geiregat, Z. Zanolli, and D. Vanmaekelbergh, "Role of surface bands in the photogeneration, cooling, and recombination of charge carriers in two-dimensional Bi_2Se_3," *ACS Nano* **19**, 17261-17272 (2025).

[2] J. R. Moes, J. F. Vliem, P. M. M. C. de Melo, T. C. Wigmans, A. R. Botello-Méndez, R. G. Mendes, E. F. van Brenk, I. Swart, L. Maisel Licerán, H. T. C. Stoof, C. Delerue, Z. Zanolli, and D. Vanmaekelbergh, "Characterization of the Edge States in Colloidal Bi_2Se_3 Platelets," *Nano Lett.* **24**, 5110-5116 (2024).

[3] P.-H. Tan, W. P. Han, W. J. Zhao, Z. H. Wu, K. Chang, H. Wang, Y. F. Wang, N. Bonini, N. Marzari, N. Pugno, *et al.*, "The Shear Mode of Multilayer Graphene," *Nat. Mater.* **11**, 294-300 (2012).

[4] Y. Zhao, X. Luo, J. Zhang, J. Wu, X. Bai, M. Wang, J. Jia, H. Peng, Z. Liu, S. Y. Quek, and Q. Xiong, "Interlayer vibrational modes in few-quintuple-layer Bi_2Te_3 and Bi_2Se_3 two-dimensional crystals: Raman spectroscopy and first-principles studies," *Phys. Rev. B* **90**, 245428 (2014).

[5] X. Chen, H. D. Zhou, A. Kiswandhi, I. Miotkowski, Y. P. Chen, P. A. Sharma, A. L. Lima Sharma, M. A. Hekmaty, D. Smirnov, and Z. Jiang, "Thermal expansion coefficients of Bi_2Se_3 and Sb_2Te_3 crystals from 10 K to 270 K," *Appl. Phys. Lett.* **99**, 261912 (2011).
